# Long-term immunogenicity and safety of a non-typeable *Haemophilus influenzae*-*Moraxella catarrhalis* vaccine: 4-year follow-up of a phase 1 multicentre trial

**DOI:** 10.1016/j.jvacx.2021.100124

**Published:** 2021-11-03

**Authors:** Philippe De Smedt, Geert Leroux-Roels, Corinne Vandermeulen, Annaelisa Tasciotti, Gennaro Di Maro, Marie Dozot, Daniela Casula, Margherita Annaratone, Daniele Riccucci, Ashwani Kumar Arora

**Affiliations:** a Centre for the Evaluation of Vaccination, Vaccine and Infectious Disease Institute, University of Antwerp, Antwerp, Belgium; b Centre for Vaccinology, Ghent University and Ghent University Hospital, Ghent, Belgium; c Leuven University Vaccinology Centre, Department of Public Health and Primary Care, KU Leuven, Leuven, Belgium; d GSK, Siena, Italy; e GSK, Rixensart, Belgium

**Keywords:** Acute exacerbation, Antibody persistence, Clinical trial, COPD, *Haemophilus influenzae*, *Moraxella catarrhalis*, AECOPD, acute exacerbations of chronic obstructive pulmonary disease, ANCOVA, analysis of covariance, AS01_E_, Adjuvant System AS01_E_, containing 3-*O*-desacyl-4′-monophosphoryl lipid A, QS-21 (*Quillaja saponaria* Molina, fraction 21) and liposome, CI, confidence interval, COPD, chronic obstructive pulmonary disease, ELISA, enzyme-linked immunosorbent assay, EU, enzyme-linked immunosorbent assay units, GMC, geometric mean concentration, GMR, geometric mean ratio, LLOQ, lower limit of quantification, Mcat, *Moraxella catarrhalis*, MPL, 3-*O*-desacyl-4′-monophosphoryl lipid A, NTHi, non-typeable *Haemophilus influenzae*, PD, protein D, PE, protein E, PilA, Pilin A, pIMD, potential immune-mediated disease, QS-21, *Quillaja saponaria* Molina, fraction 21, SAE, serious adverse event, UspA2, ubiquitous surface protein A2

## Abstract

A multicomponent vaccine has been developed to reduce the frequency of acute exacerbations of COPD associated with non-typeable *Haemophilus influenzae* (NTHi) and *Moraxella catarrhalis* (Mcat) infections, containing NTHi (PD and PE-PilA) and Mcat (UspA2) surface proteins. In a randomised, observer-blind, placebo-controlled study with two steps (NCT02547974), the investigational vaccine had good immunogenicity and no safety concerns were identified. In step 2, 90 adults aged 50–71 years with smoking history received two doses 60 days apart of one of two AS01_E_-adjuvanted formulations containing 10 µg of each antigen (10–10-AS01) or 10 µg NTHi antigens and 3.3 µg UspA2 (10–3-AS01), or placebo. Long-term persistence of antigen-specific humoral antibodies was assessed in 81 participants during 3 years of follow-up after the initial 14-month study (NCT03201211).

Antigen-specific antibody concentrations were measured in blood samples taken every 6 months. Safety monitoring evaluated serious adverse events (SAEs) and potential immune-mediated disease (pIMD).

Immune responses against NTHi antigens persisted up to 4 years post-vaccination. For PD, PE and PilA, at each follow-up time point, adjusted antibody geometric mean concentrations (GMCs) were higher (non-overlapping 95% confidence intervals [CIs]) in the vaccine groups versus placebo and versus pre-vaccination. Antibody GMC point estimates were higher with 10–3-AS01 than with 10–10-AS01. For UspA2, 95% CIs included 1 for GMC ratios of 10–10-AS01 or 10–3-AS01 to placebo at each time point. During follow-up, SAEs were reported in nine (11.1%) participants, one of which was fatal (lung cancer, 607 days after second 10–10-AS01 dose). One non-serious pIMD, trigeminal neuralgia, was reported 771 days after second 10–3-AS01 dose. The SAEs and pIMD were considered not related to vaccination.

Immune responses against NTHi antigens persisted for 4 years after two-dose vaccination with the investigational NTHi-Mcat vaccine. There was no persistent response against the Mcat antigen. No safety concerns were identified during the long-term follow-up.

## Highlights

• Older adults with smoking history received two doses of combined NTHi-Mcat vaccine.
• We evaluated antibody persistence during 4 years of follow-up after vaccination.
• Immune responses against the NTHi protein antigens persisted up to 4 years.
• There was no persistent immune response against the Mcat antigen.
• No safety concerns were identified during the long-term follow-up period.

## Introduction

Chronic obstructive pulmonary disease (COPD) is the third leading cause of death globally [1], with an estimated prevalence of 12% in people aged 30 years or more [2]. Acute exacerbations of COPD (AECOPD) are periods of worsened respiratory symptoms, beyond that seen with day-to-day variation, that increase the risk of myocardial infarction, stroke, pulmonary embolism and death [3]. No vaccine is approved for the prevention of AECOPD, although influenza and pneumococcal vaccines, which are routinely recommended to COPD patients [4], may have some effect on the frequency of exacerbations [5].

Bacterial infection is frequently associated with AECOPD, most commonly non-typeable *Haemophilus influenzae* (NTHi), *Moraxella catarrhalis* (Mcat) and *Streptococcus pneumoniae* infections [6], [7], [8], [9]. Targeting the major bacterial species associated with AECOPD may be a viable strategy for vaccine development. There is evidence that NTHi and Mcat can act as co-pathogens in respiratory tract infections and COPD, as indicated by protection of NTHi from complement-mediated killing via complement resistance factors on outer membrane vesicles produced by Mcat [10]. Increased resistance to antibiotics and host clearance also appears to be promoted by NTHi and Mcat co-infection [11], [12].

An adjuvanted multicomponent vaccine has been developed to reduce the frequency of moderate and severe AECOPD associated with NTHi and Mcat. The investigational NTHi-Mcat vaccine contains four surface proteins involved in the virulence mechanisms of both bacterial pathogens [13]. Three are from NTHi, a free recombinant protein D (PD) and a recombinant fusion protein combining protein E and Pilin A (PE-PilA), and the fourth from Mcat, ubiquitous surface protein A2 (UspA2). Evidence from animal studies suggest anti-PD antibodies have opsonic activity [14] and protect against *H. influenzae* infection [15], while antibodies generated by the PE-PilA fusion protein inhibit the binding of PE to vitronectin (which may protect the bacterium from complement attack [16], [17]) and inhibit the formation of NTHi biofilms [18], as previously described for anti-PilA antibodies [19]. Anti-UspA2 antibodies significantly reduced the lung bacterial load in mice challenged with homologous or heterologous Mcat strains [20] and have been shown to be bactericidal and cross-reactive [20], [21]. A vaccine formulation containing the NTHi proteins had an acceptable safety and reactogenicity profile and induced antigen-specific immune responses in phase 1 studies of healthy 18–40 year-olds and current and former smokers aged 50–70 years [22]. The population group of adults with smoking history was chosen to immunologically match the COPD population, with evidence suggesting that alterations in the immune system start early in smokers, before COPD is diagnosed [23], [24], [25]. NTHi vaccine formulations that included the Adjuvant System AS01_E_ [26] produced the highest humoral and cellular immune responses in older adults [22]. A phase 2 study of the adjuvanted NTHi vaccine in adults with COPD showed no safety concerns and good immunogenicity [27].

In the first clinical assessment of the safety, reactogenicity and immunogenicity of the investigational NTHi-Mcat vaccine, two doses were given 60 days apart and the study was conducted in two steps [13]. In step 1, healthy adults aged 18–40 years received a non-adjuvanted vaccine formulation or placebo. In step 2, older adults with a smoking history received one of two AS01_E_-adjuvanted formulations (one containing 10 µg PD, 10 µg PE-PilA and 10 µg UspA2 and the other 10 µg PD, 10 µg PE-PilA and 3.3 µg UspA2) or placebo. No safety concerns were identified with the NTHi-Mcat vaccine and the formulation containing 3.3 µg UspA2 induced the best humoral response against NTHi antigens [13]; this response was consistent with that induced by the adjuvanted NTHi vaccine in the phase 2 study of patients with COPD [27]. The anti-UspA2 immune response was moderate and transient with both formulations. In the present study, we assessed the long-term persistence of antigen-specific humoral antibodies in the groups included in step 2 of the initial study. In a follow-up period of 3 years (i.e. up to 4 years after the second vaccine dose), safety monitoring also continued, specifically evaluation of serious adverse events (SAEs) and potential immune-mediated disease (pIMD).

## Methods

### Study design and participants

This was a 3-year open-label follow-up study (ClinicalTrials.gov identifier: NCT03201211) of a 14-month phase 1, randomised, observer-blind, placebo-controlled study conducted in Belgium between August 2015 and March 2017 (NCT02547974). The methods of the phase 1 study were described previously [13] and a study summary is available at www.gsk-studyregister.com (study identifier, 204913). Briefly, participants were randomised to receive two vaccine formulation doses 60 days apart. There were two steps: in step 1, 30 healthy adults aged 18–40 years received a non-adjuvanted vaccine formulation containing 10 µg of each NTHi antigen and 10 µg of UspA2 antigen per dose or placebo. In step 2, 90 healthy adults aged 50–71 years with a smoking history of at least 10 pack-years received an AS01_E_-adjuvanted formulation containing either 10 µg of each antigen (10–10-AS01) or 10 µg of each NTHi antigen and 3.3 µg of Mcat UspA2 (10–3-AS01), or placebo. The NTHi antigens were described previously [22]. AS01_E_ is an Adjuvant System containing 3-*O*-desacyl-4′-monophosphoryl lipid A (MPL), QS-21 (*Quillaja saponaria* Molina, fraction 21; licensed by GSK from Antigenics LLC, a wholly owned subsidiary of Agenus Inc., a Delaware, USA corporation) and liposome (25 μg MPL and 25 µg QS-21) [26].

In this follow-up study, the primary objective was to evaluate the persistence of humoral antibodies in the groups of participants included in step 2 of the initial study, up to 3 years after the last study visit. The last study visit of step 2 occurred 14 months after the study start and 12 months after the second vaccine dose. The secondary objective was to assess the long-term safety of the investigational NTHi-Mcat vaccine by monitoring the occurrence of SAEs and pIMD over the 3-year follow-up period. Eligible participants had participated in step 2 of the initial study, had received two doses, and were able to return for follow-up visits. Participants were excluded if they received or were scheduled to receive any investigational or non-registered vaccine or drug during the study. Other exclusion criteria included receipt of an immune-modifying drug, immunoglobulins or blood products during the study period, and any acute or chronic condition that could interfere with the results of the study.

The study was conducted in accordance with the Declaration of Helsinki and Good Clinical Practice. The protocols and associated documents were reviewed and approved by an independent ethics committee. All participants provided written informed consent before study entry.

### Antibody measurement

Immunoglobulin G antibody concentrations to each vaccine antigen were measured by enzyme-linked immunosorbent assay (ELISA), developed by GSK Biologicals and validated (for PD) or qualified (qualification in parallel with study conduct for PE, PilA and UspA2) on blood samples collected every 6 months during the 3-year follow-up, which started 14 months after the start of the study (12 months since the second vaccine dose), i.e. at 20, 26, 32, 38, 44 and 50 months from the start of the study. Sera were stored at −20 °C until assayed. Standardised procedures and in-house-made reference serum were used for each assay. The cut-off of the assays (i.e. lower limit of quantification, LLOQ) was 153 ELISA units (EU)/mL, 25 EU/mL, 16 EU/mL and 38 EU/mL for anti-PD, anti-PE, anti-PilA and anti-UspA2, respectively, for the first two time points, and 153 EU/mL, 16 EU/mL, 8 EU/mL and 28 EU/mL, respectively, for the remaining time points (post-qualification).

### Safety analyses

During the entire follow-up study, the occurrence of SAEs was recorded, defined as any untoward medical occurrence that resulted in death, was life-threatening, required hospitalisation or prolongation of hospitalisation, resulted in disability or incapacity, or was a congenital anomaly or birth defect in the child of a subject. Data on pIMD, which included autoimmune and other inflammatory or neurological disorders of interest [28], were also recorded regardless of seriousness. Investigators or site staff were responsible for detecting, documenting and reporting events meeting the criteria of a SAE or pIMD. Safety oversight was provided by a safety review team through ongoing routine safety monitoring.

### Statistical analysis

It was planned to enrol all eligible participants from step 2 of the initial study [13]. The persistence of humoral antibodies was analysed in the per-protocol cohort for immunogenicity, which consisted of all eligible participants who were compliant with study procedures and had at least one assay result for at least one of the follow-up time points.

Descriptive exploratory analyses characterised the differences between groups in humoral immune response, as measured by ELISA geometric mean concentration (GMC). For analysis purposes, samples with concentrations that fall below the LLOQ were assigned a value of half the LLOQ to calculate the GMC. The difference between groups in antibody GMC was evaluated by calculating the 95% confidence intervals (CIs) of the ratio of antibody GMCs between groups, using a one-way analysis of covariance (ANCOVA) model on the log_10_ transformation of antibody concentrations. The ANCOVA model included group category as fixed effect and the baseline antibody log_10_ concentration (before the first vaccine dose in the initial study) as covariate. Geometric mean ratios (GMRs) with 95% CIs were calculated to describe the mean change in antibody concentration at a specific time point with respect to the pre-vaccination antibody concentration for each group. GMRs were calculated using a one-way analysis of variance model on the ratio between the log_10_ antibody concentration at a specific time point and the baseline log_10_ antibody concentration, with group category as a fixed effect. Any differences between groups or time points should be interpreted with caution, as no adjustment for multiplicity was performed when computing the CIs.

The safety analysis was performed on all enrolled participants. Statistical analyses were performed using Statistical Analysis System Version 9.4 on Life Science Analytics Framework 4.3 (SAS Institute Inc., Cary, NC, USA).

## Results

### Study population

Eighty-one healthy adults with a smoking history were enrolled in this follow-up study (27 in the 10–10-AS01 group, 26 in the 10–3-AS01 group and 28 in the placebo group). Immunogenicity results were available for at least one time point for all enrolled participants. Demographic characteristics were similar between groups (Table 1). All participants were white (European heritage).

**Table 1:** Demographic characteristics of participants enrolled in the follow-up study.

Characteristic	10–10-AS01 (N = 27)	10–3-AS01 (N = 26)	Placebo (N = 28)
Age (years) at dose 1, mean (SD)	59.7 (6.3)	59.0 (5.9)	58.2 (6.5)
Age group (years), n (%)			
50–59	14 (51.9)	15 (57.7)	18 (64.3)
60–70	13 (48.1)	11 (42.3)	10 (35.7)
Male sex, n (%)	15 (55.6)	14 (53.8)	19 (67.9)
Smoking status, n (%)			
Current smoker	10 (37.0)	8 (30.8)	10 (35.7)
Former smoker	17 (63.0)	18 (69.2)	18 (64.3)

10–10-AS01, group that received vaccine containing 10 µg of each non-typeable *Haemophilus influenzae* (NTHi) antigen and 10 µg of *Moraxella catarrhalis* (Mcat) antigen with AS01_E_; 10–3-AS01, group that received vaccine containing 10 µg of each NTHi antigen and 3.3 µg of Mcat antigen with AS01_E_. N, number of participants; n, number of participants in a specific category; SD, standard deviation.

### Immunogenicity

**Fig. 1:**
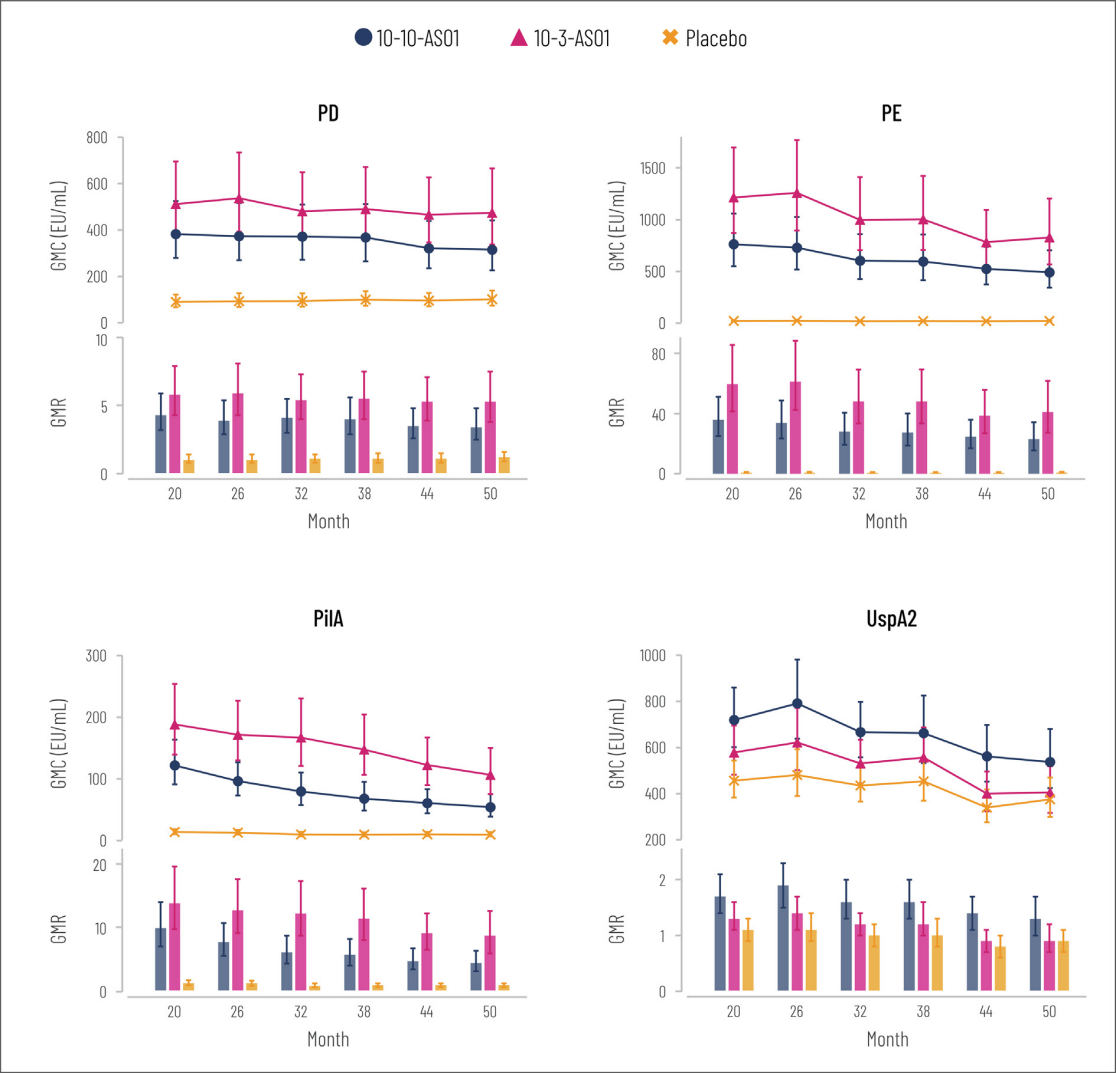
Geometric mean concentrations (GMCs with 95% CIs, adjusted for baseline antibody log_10_ concentrations) and geometric mean ratios (GMRs with 95% CIs) of log_10_ antibody concentrations at each time point versus pre-vaccination during follow-up (per-protocol immunogenicity cohort). Months 20, 26, 32, 38, 44 and 50 equate to 18, 24, 30, 36, 42 and 48 months after the second vaccine dose. Number of participants with available results at each time point: between 24 and 27 in 10–10-AS01 group, 23 and 26 in 10–3-AS01 group, 26 and 28 in placebo group. EU, enzyme-linked immunosorbent assay units; PD, protein D; PE, protein E; PilA, Pilin A; UspA2, ubiquitous surface protein A2; 95% CI, 95% confidence interval; 10–10-AS01, group that received vaccine containing 10 µg of each non-typeable *Haemophilus influenzae* (NTHi) antigen and 10 µg of *Moraxella catarrhalis* (Mcat) antigen with AS01_E_; 10–3-AS01, group that received vaccine containing 10 µg of each NTHi antigen and 3.3 µg of Mcat antigen with AS01_E_.

**Fig. 2:**
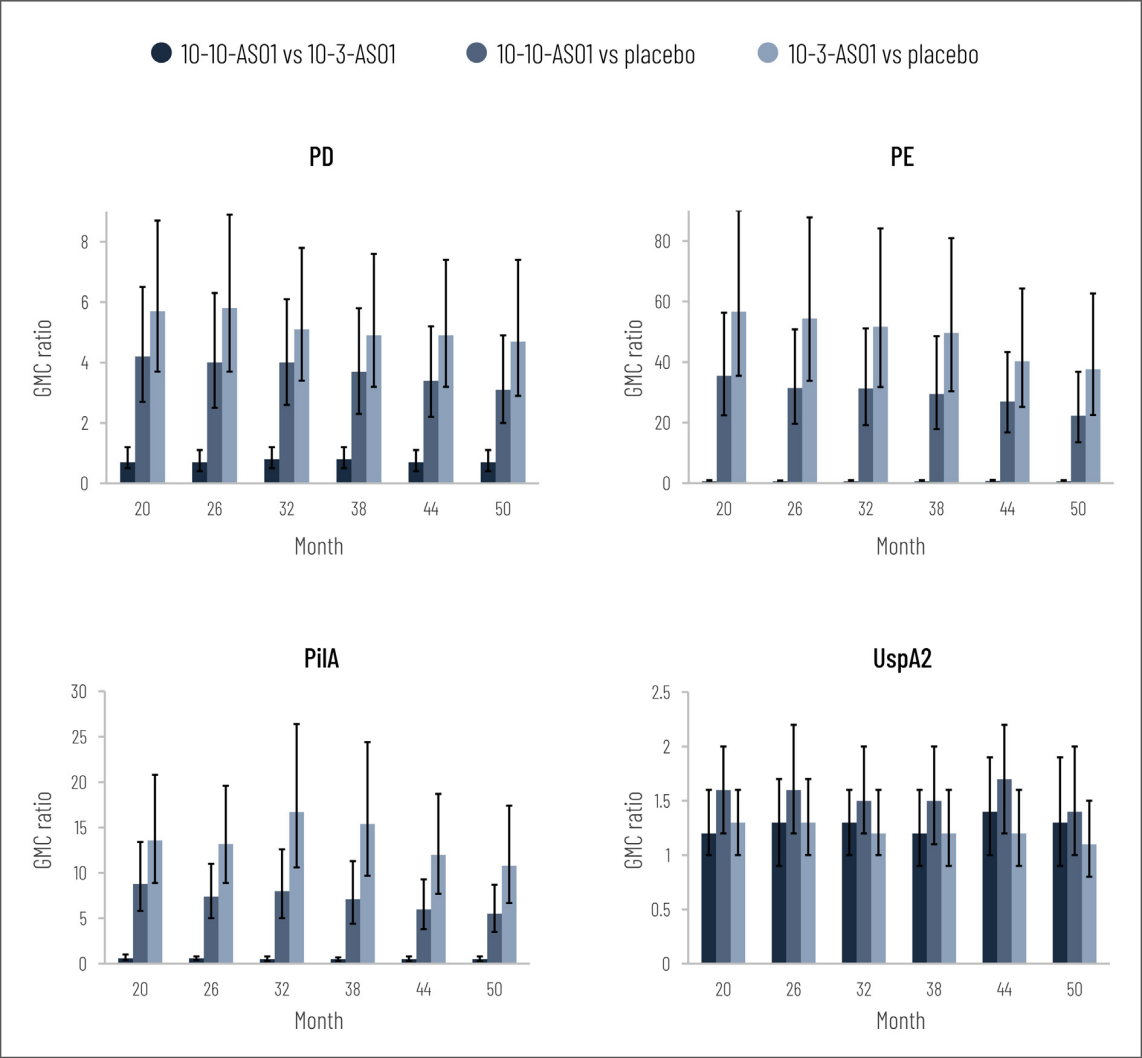
Geometric mean concentration ratios (GMC ratios with 95% CIs, adjusted for baseline antibody log_10_ concentrations) between groups at each time point during follow-up (per-protocol immunogenicity cohort). Months 20, 26, 32, 38, 44 and 50 equate to 18, 24, 30, 36, 42 and 48 months after the second vaccine dose. Number of participants with available results at each time point: between 24 and 27 in 10–10-AS01 group, 23 and 26 in 10–3-AS01 group, 26 and 28 in placebo group. PD, protein D; PE, protein E; PilA, Pilin A; UspA2, ubiquitous surface protein A2; 95% CI, 95% confidence interval; 10–10-AS01, group that received vaccine containing 10 µg of each non-typeable *Haemophilus influenzae* (NTHi) antigen and 10 µg of *Moraxella catarrhalis* (Mcat) antigen with AS01_E_; 10–3-AS01, group that received vaccine containing 10 µg of each NTHi antigen and 3.3 µg of Mcat antigen with AS01_E_.

Immune responses against the NTHi antigens (PD, PE and PilA) persisted during the 3-year follow-up period after initial study completion (Fig. 1; supplementary Fig. S1). At each follow-up time point, adjusted antibody GMCs against each NTHi antigen were higher in the vaccine groups versus placebo, as indicated by non-overlapping 95% CIs (Fig. 1). GMC ratios for vaccine group versus placebo were higher than or equal to 3.1 (95% CI: 2.0–4.9) for PD, 22.3 (13.5–36.8) for PE and 5.5 (3.5–8.7) for PilA (Fig. 2). In the vaccinated groups, GMRs at each time point were higher than or equal to 3.4 (95% CI: 2.5–4.8) for PD, 23.2 (15.7–34.3) for PE and 4.4 (3.1–6.3) for PilA (Fig. 1). Antibody GMC point estimates for the NTHi antigens were higher in the 10–3-AS01 group than in the 10–10-AS01 group at each time point (Fig. 1).

For the Mcat antigen, UspA2, GMC point estimates were higher in the vaccine groups at each follow-up time point compared to placebo (Fig. 1, supplementary Fig. S1). GMRs in the vaccinated groups were between 1.2 and 1.9 at each time point, apart from in the 10–3-AS01 group at months 44 and 50 (GMR 0.9); all GMR 95% CIs included 1 (Fig. 1). Antibody GMC ratios for 10–10-AS01 versus placebo and 10–3-AS01 versus placebo were 1.7 (95% CI: 1.2–2.2) or lower (Fig. 2).

### Safety

At least one SAE was reported in nine (11.1%) participants: five reported nine SAEs in the 10–10-AS01 group, two reported two SAEs in 10–3-AS01 group, and two reported two SAEs in the placebo group (Table 2). No SAE category (preferred term) was reported more than once (Table 2) and none of the SAEs were assessed as related to vaccination. Eight participants experienced unsolicited adverse events leading to hospitalisation: four in the 10–10-AS01 group, two in the 10–3-AS01 group and two in the placebo group.

**Table 2:** Participants with at least one serious adverse event (SAE), by Medical Dictionary for Regulatory Activities (MedDRA) preferred term.

MedDRA preferred term	Number of participants (percentage; 95% CI)		
	10–10-AS01 (N = 27)	10–3-AS01 (N = 26)	Placebo (N = 28)
At least one SAE	5 (18.5; 6.3–38.1)*	2 (7.7; 0.9–25.1)	2 (7.1; 0.9–23.5)
Humerus fracture	1 (3.7; 0.1–19.0)	0	0
Tibia fracture	0	0	1 (3.6; 0.1–18.3)
Tendon rupture	1 (3.7; 0.1–19.0)	0	0
Post-procedural fever	1 (3.7; 0.1–19.0)	0	0
Lung neoplasm malignant	1 (3.7; 0.1–19.0)	0	0
Schwannoma	1 (3.7; 0.1–19.0)	0	0
Ileus	1 (3.7; 0.1–19.0)	0	0
Ileus paralytic	1 (3.7; 0.1–19.0)	0	0
Intestinal obstruction	1 (3.7; 0.1–19.0)	0	0
Post-operative wound infection	1 (3.7; 0.1–19.0)	0	0
Spinal stenosis	0	1 (3.8; 0.1–19.6)	0
Ischaemic stroke	0	1 (3.8; 0.1–19.6)	0
Nephrolithiasis	0	0	1 (3.6; 0.1–18.3)

10–10-AS01, group that received vaccine containing 10 µg of each non-typeable *Haemophilus influenzae* (NTHi) antigen and 10 µg of *Moraxella catarrhalis* (Mcat) antigen with AS01_E_; 10–3-AS01, group that received vaccine containing 10 µg of each NTHi antigen and 3.3 µg of Mcat antigen with AS01_E__;_ N, number of participants for each study group; 95% CI, exact 95% confidence interval.

* Post-procedural fever, Schwannoma and post-operative wound infection reported in a single subject; ileus, ileus paralytic and intestinal obstruction reported in a single subject.

There was one death, which was due to lung cancer and occurred in the 10–10-AS01 group 607 days (approximately 20 months) after receiving the second vaccine dose. This was considered not related to study vaccination. One non-serious pIMD, trigeminal neuralgia, was reported in the 10–3-AS01 group in an individual 771 days (over 2 years) after the second vaccination. The pIMD was of moderate intensity, lasted 37 days, and the participant recovered fully. This event was not considered to be causally related to vaccination.

## Discussion

This is the first report of the long-term persistence of antibodies induced by vaccination against NTHi antigens, PD and PE-PilA, and Mcat antigen, UspA2. The results show the adjuvanted NTHi-Mcat vaccine formulations induced persistent immune responses against the NTHi antigens up to 4 years after the second vaccine dose, with the 10–3-AS01 formulation inducing the best humoral responses. There was no persistent response against UspA2. These findings are consistent with those from the initial 12-month follow-up period [13].

The good long-term persistence of antibody against NTHi antigens is possibly due to the adjuvant used in the vaccine, AS01_E_. The liposome-based AS01 Adjuvant System has been shown to enhance adaptive immune responses against antigens from pathogens causing complex diseases including malaria, hepatitis and shingles [26], [29]. There is also evidence that inclusion of an Adjuvant System in the vaccine can produce persistent immune responses against *H. influenzae*. A study of an investigational vaccine containing three protein antigens, including *H. influenzae* PD, reported persistent anti-PD antibodies in healthy adults 1 year after two vaccine doses and higher responses when the vaccine was administered with an Adjuvant System containing α-tocopherol and squalene in an oil-in-water emulsion, AS03 [30].

In the initial study, there was a moderate but transient specific response against the Mcat antigen [13]. In the subsequent 3-year follow-up, all GMC ratio and GMR 95% CIs included 1 for each UspA2 assessment, indicating no differences between groups or differences from baseline. This lack of persistent immune response could be caused by several factors. It may have been due to natural boosting by repetitive exposure or colonisation by Mcat, and the conserved nature of UspA2, allowing successful detection before vaccination in the ELISA. The concentration of anti-UspA2 antibodies before vaccination was relatively high in all groups (384.1–572.5 EU/mL in the initial study [13]; see also Fig. S1), suggesting strong natural exposure. An inverse association between pre-vaccination titre and vaccine response has been reported in studies of other vaccines, such as influenza and respiratory syncytial virus vaccines, and oral cholera vaccines [31], [32], [33]. Also, it is clear from individual subject listings that the immune response against UspA2 was highly variable (data not shown). While the UspA2 protein induces a bactericidal and cross-reactive immune response [21], recent evidence indicates that it has a variable sequence and structure [34], [35], which may have an impact on antibody recognition and binding.

As reported in the initial 14-month study [13], in the 4-year follow-up, the vaccine formulation containing 3.3 µg UspA2 induced higher specific responses (higher antibody GMC point estimates) against the NTHi antigens than the formulation containing 10 µg UspA2. Conversely, anti-UspA2 point estimates were lower with the 3.3 µg UspA2 formulation than with the 10 µg formulation. This is suggestive of immunological interference, with a higher quantity of UspA2 leading to a higher response against the Mcat antigen but lower response against NTHi antigens, and the formulation with lower UspA2 quantity inducing a lower UspA2 response but better responses against NTHi antigens.

No safety concerns were identified during the long-term follow-up period. Thirteen SAEs were reported over 3 years, with no SAE (preferred term) reported more than once, and one non-serious pIMD was reported, occurring in the 10–3-AS01 group. One death occurred in the 10–10-AS01 group. All were assessed as not causally related to vaccination.

This study is limited by its open-label design and small sample size. Also, the long-term persistence of antibodies may differ in the target population for the vaccine (COPD patients). A strength of the study was the enrolment of most participants (81) from the cohort of 90 included in step 2 of the initial study.

In conclusion, in the 4-year period after two-dose vaccination with the investigational NTHi-Mcat vaccine, there was long-term persistence of immune responses against the NTHi protein antigens. This was not observed for the Mcat antigen, UspA2, although a decline in anti-UspA2 immune response was already present at the end of the initial 14-month study. No safety concerns were identified.

## Declaration of Competing Interest

The authors declare the following financial interests/personal relationships which may be considered as potential competing interests: PDS declares no financial or non-financial relationships and activities and no conflicts of interest. GL-R reports a grant paid by the GSK group of companies for the conduct of this study and consulting fees paid by the GSK group of companies in the context of clinical trials conducted in general. CV reports a grant paid to her employer by the GSK group of companies during the conduct of this study, and grants paid to her employer by MSD and Pfizer outside the submitted work. AT, GDM, MD, DC, MA, DR and AKA are employees of the GSK group of companies. MD holds shares in the GSK group of companies and is married to an employee of the GSK group of companies who holds shares in it. GL-R, CV, AT, GDM, MD, DC, MA, DR and AKA declare no other financial or non-financial relationships and activities.
