# Supplementary material for: Long-term immunogenicity and safety of a non-typeable Haemophilus influenzae-Moraxella catarrhalis vaccine: 4-year follow-up of a phase 1 multicentre trial
Source: Vaccine X. 2021 Nov 3;9:100124. doi: 10.1016/j.jvacx.2021.100124 (PMC8600057; doi:10.1016/j.jvacx.2021.100124)
Supplement: Supplementary data 1 [file mmc1.docx]

# Supplementary material

**Fig. S1.** Geometric mean concentrations (GMCs with 95% CIs, adjusted for baseline antibody log_10_ concentrations) during follow-up after vaccination with investigational NTHi-Mcat vaccine at months 0 and 2 (per-protocol immunogenicity cohort). Results for the initial 14-month study (to left of grey vertical line) were calculated for participants enrolled in follow-up study.


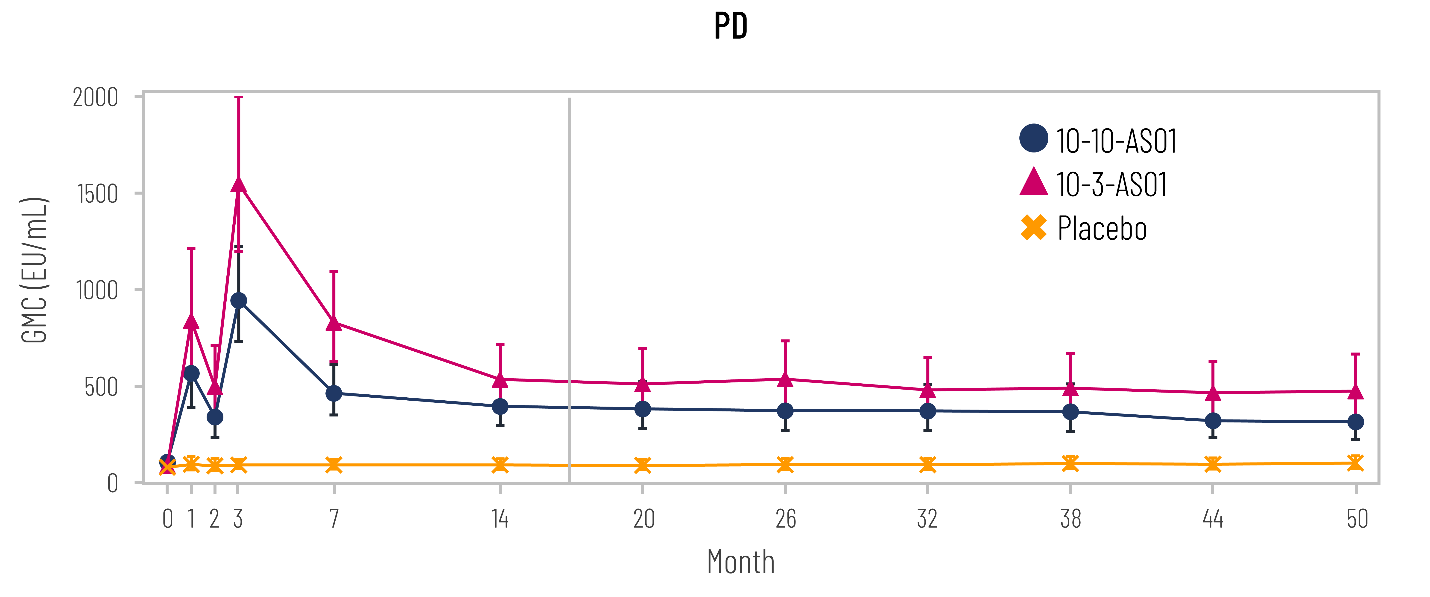

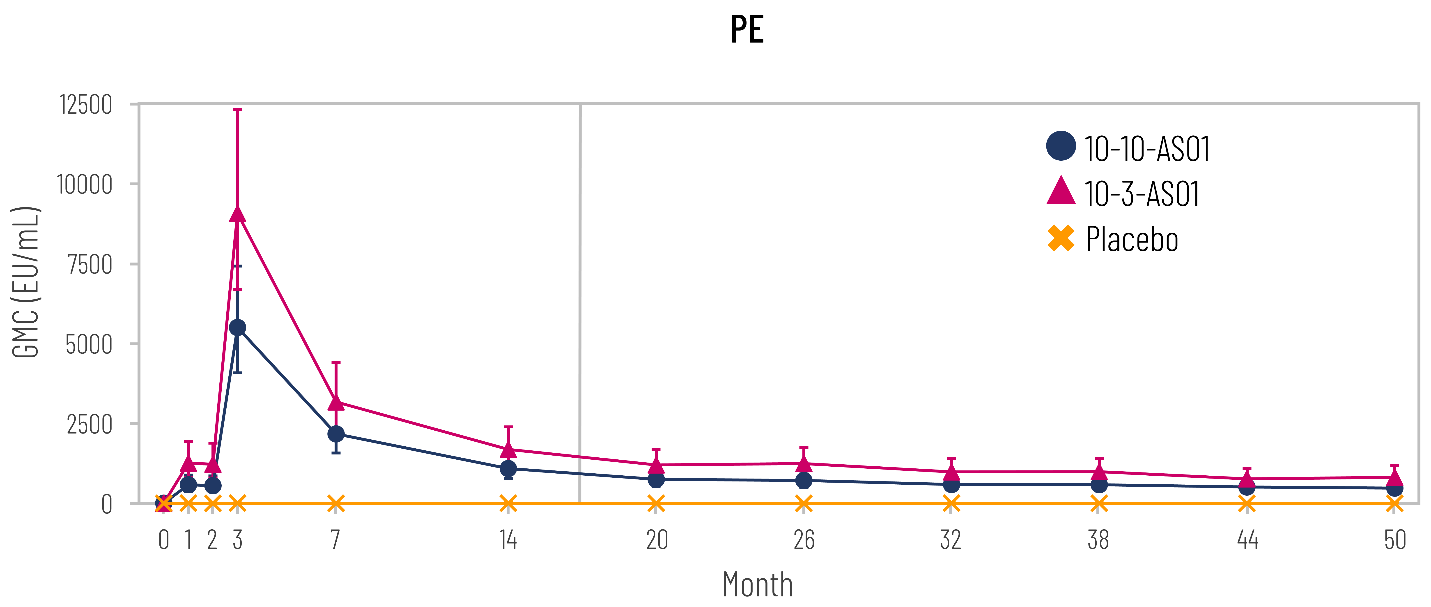


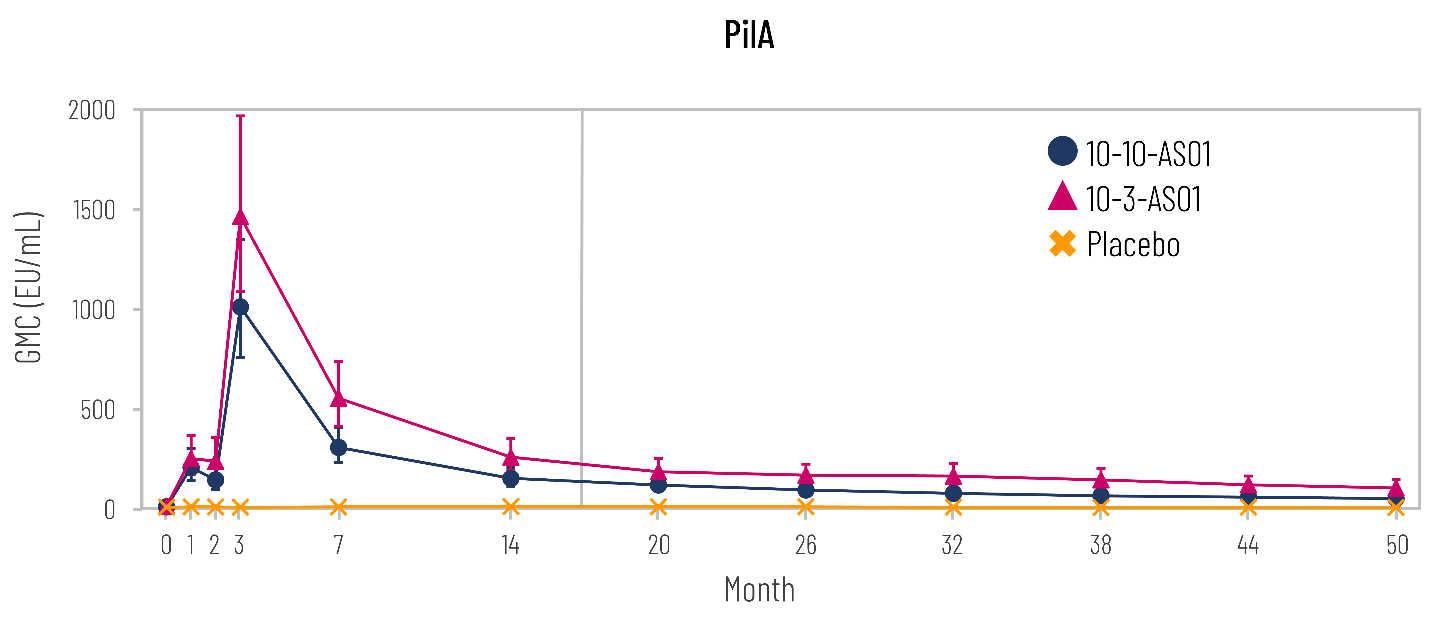

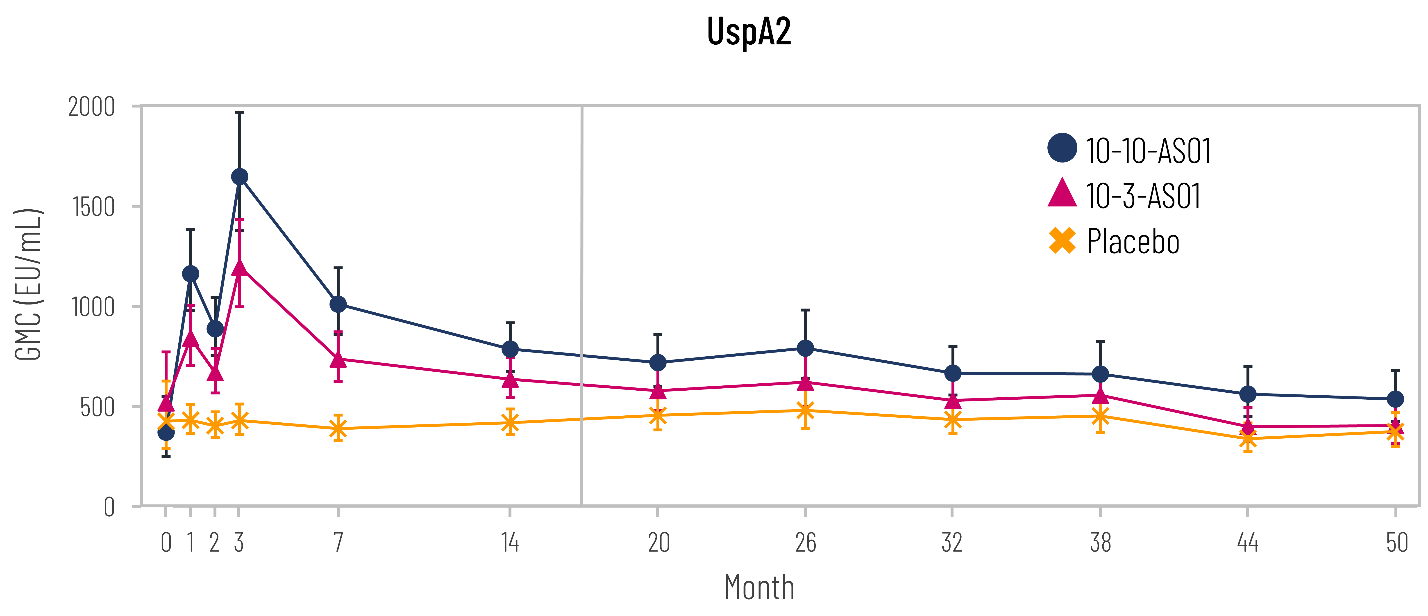


Months 3, 7, 14, 20, 26, 32, 38, 44 and 50 equate to 1, 5, 12, 18, 24, 30, 36, 42 and 48 months after the second vaccine dose. EU, enzyme-linked immunosorbent assay units; PD, protein D; PE, protein E; PilA, Pilin A; UspA2, ubiquitous surface protein A2; 95% CI, 95% confidence interval; 10-10-AS01, group that received vaccine containing 10 µg of each non-typeable *Haemophilus influenzae* (NTHi) antigen and 10 µg of *Moraxella catarrhalis* (Mcat) antigen with AS01_E_; 10-3-AS01, group that received vaccine containing 10 µg of each NTHi antigen and 3.3 µg of Mcat antigen with AS01_E_.
